# Biofertilizer and Antifungal Potential of *Streptomyces* spp. in Greenhouse-Grown Tomato Plants (*Solanum lycopersicum* Mill.)

**DOI:** 10.3390/plants15121766

**Published:** 2026-06-08

**Authors:** Erika Santamaría-Pérez, Ana Vélez-Pardo, Alejandro Acosta-González, Carlos Jiménez-Junca, Fernando Bautista-Pinzón, Luis E. Díaz, Natalia Conde-Martínez

**Affiliations:** 1 Master’s Program in Process Design and Management, Faculty of Engineering, Universidad de La Sabana, Kilometer 7, Autopista Norte, Chía 250001, Colombia; erikasanpe@unisabana.edu.co (E.S.-P.); anavepar@unisabana.edu.co (A.V.-P.); josebapi@unisabana.edu.co (F.B.-P.); 2 Bioprospecting Research Group (GIBP), Faculty of Engineering, Universidad de La Sabana, Campus del Puente del Común, Kilometer 7, Autopista Norte de Bogotá, Chía 250001, Colombia; alejandro.acosta1@unisabana.edu.co (A.A.-G.); carlos.jimenez@unisabana.edu.co (C.J.-J.); 3 Independent Researcher, Bogotá 111311, Colombia; lediazb@gmail.com

**Keywords:** *Streptomyces* spp., plant growth-promoting (PGP), *Fusarium oxysporum*, tomato (*Solanum lycopersicum*), greenhouse conditions

## Abstract

*Fusarium oxysporum* f. sp. *lycopersici* is one of the most destructive soilborne pathogens affecting tomato production, reducing plant growth and yield and highlighting the need for sustainable management alternatives. *Streptomyces* spp. are promising microbial candidates due to their ability to combine antifungal activity with plant growth promotion characteristics. The objective of this study was to evaluate the biofertilizer and antifungal potential of *Streptomyces* spp. in Chonto tomato (*Solanum lycopersicum* Mill.) under greenhouse conditions. Seventy actinobacterial strains were screened in vitro against *F. oxysporum*, and eight exhibited significant antagonistic activity. Based on antagonistic activity, enzymatic profile, cytotoxicity, and plant growth-promoting potential, strains 1B260 and 445 were selected for greenhouse assays. Strain 1B260 achieved 43.5% mycelial growth inhibition and showed the highest phosphate-solubilizing capacity (420 µg/mL), while both strains displayed proteolytic and cellulolytic activity, low cytotoxicity in human skin cell lines (HaCaT and HDFa), nitrogen fixation, and ammonia production. In greenhouse assays under non-infected conditions, 1B260 showed the most consistent biofertilizer effect, promoting stem elongation. Under pathogen pressure, strain 445 improved plant performance compared to the infected control. Overall, strains 1B260 and 445 exhibited complementary roles in tomato crop management, highlighting the potential of multifunctional *Streptomyces* inoculants for sustainable biofertilization and biocontrol strategies.

## 1. Introduction

Tomato (*Solanum lycopersicum*) ranks as the leading vegetable crop worldwide, accounting for approximately 16% of total global vegetable production [1], with 192.3 million tons harvested from 5.41 million hectares in 2023, according to the Food and Agriculture Organization of the United Nations (FAOSTAT). In Colombia, the Chonto type constitutes a major horticultural commodity, producing nearly one million tons annually across more than 16,000 hectares and serving as a principal variety for fresh consumption [2,3]. Beyond their high fiber content (11.44 g/100 g), antioxidants (54.74 mg/100 g), micronutrients (35.18 mg/100 g), and vitamins (67.79 mg/100 g), they are also traditionally used in certain cultures to treat diarrhea and hypertension [4,5]. Tomato cultivation sustains farmer income, employment, and food security [4,6]. However, soil- and airborne fungal pathogens severely limit productivity [7,8], particularly *Fusarium oxysporum* f. sp. *lycopersici* (*Fol*), which may cause up to 100% yield loss [9,10]. Following root penetration, *Fol* colonizes xylem vessels, where mycelial proliferation and enzymatic degradation obstruct water transport, inducing wilting, chlorosis, stunting, and plant death [11].

Host susceptibility to *Fol* varies with the developmental stage of the plant, as immune competence changes with plant age [12]. Consequently, disease severity and yield reduction may depend on the phenological stage at which infection occurs [13]. Therefore, standardized phenological scales are useful for defining and comparing plant developmental stages. Since the 1980s, the BBCH scale (Biologische Bundesanstalt, Bundessortenamt, and Chemical Industry) has been used as a uniform coding system for phenological growth stages [14]. It is widely applied to characterize crop development, precocity, and maturity in relation to sowing or planting dates [15].

Nevertheless, management strategies frequently overlook the host developmental stage [16]. Historically, plant pathogen management has relied on two principal approaches: the application of synthetic chemical active ingredients, commonly referred to as conventional control, and the use of biological agents as an alternative strategy known as biological control [17]. Conventional control relies primarily on fungicides, including benomyl, carbendazim, prochloraz, fludioxonil, bromuconazole, and azoxystrobin [18]. Yet their environmental impact and the emergence of resistant strains [9] have intensified interest in biological alternatives [19]. Numerous bacterial genera exhibit protective activity [20]. There are currently 13 bacteria-based biopesticides registered in the European Union. Most of them come from *Bacillus* spp. and *Pseudomonas* spp. [21,22]. Beyond their role in disease suppression, several of these genera are also recognized as plant growth-promoting rhizobacteria (PGPR) and are widely applied as biofertilizers. Genera such as *Bacillus*, *Brevibacillus*, *Pseudomonas*, *Agrobacterium*, *Burkholderia*, *Streptomyces*, and *Thiobacillus* have been reported for this purpose [23].

Among them, the actinomycete genus *Streptomyces* has gained prominence due to its metabolic versatility and dual functionality [24]. For example, in Colombia, a biofertilizer formulated with *Streptomyces* spp. is currently available for agricultural use [25]. Species of this genus inhabit diverse environments, including the plant rhizosphere, water bodies, forest litter, river sediments, compost, and even extreme habitats [18,26]. They also synthesize antimicrobial metabolites, including kasugamycin, streptomycin, wuyiencin, streptothricins, and polyoxin [27], as well as hydrolytic enzymes such as cellulases, chitinases, lipases, and β-1,3-glucanases [2]. Growth-promoting factors such as siderophores, indole-3-acetic acid (IAA), nutrient-solubilizing activities, and 1-aminocyclopropane-1-carboxylate (ACC) deaminase-mediated ethylene modulation [11,18,28,29,30].

Given the broad spectrum of plant-beneficial activities reported for this genus, it was hypothesized that *Streptomyces* strains 445 and 1B260, isolated from the Arauca and Guaviare rivers and deposited in the Microorganism Collection of Universidad de La Sabana (USAB-BIO, RNC 243, Chía, Colombia), could act as multifunctional microbial agents by combining antifungal activity against *Fol* with plant growth-promoting effects in Chonto tomato (*Solanum lycopersicum* Mill.) seedlings under greenhouse conditions. Therefore, the objectives of this study were: (i) to evaluate the in vitro antagonistic activity of selected *Streptomyces* strains against *F. oxysporum* f. sp. *lycopersici*; (ii) to characterize enzymatic and plant growth-promoting potential associated with biocontrol and biofertilization; and (iii) to assess their effects on tomato plants growth and disease response under greenhouse conditions.

## 2. Results

### 2.1. In Vitro Assay

#### 2.1.1. Antagonistic Assay Against the Pathogen

**Figure 1:**
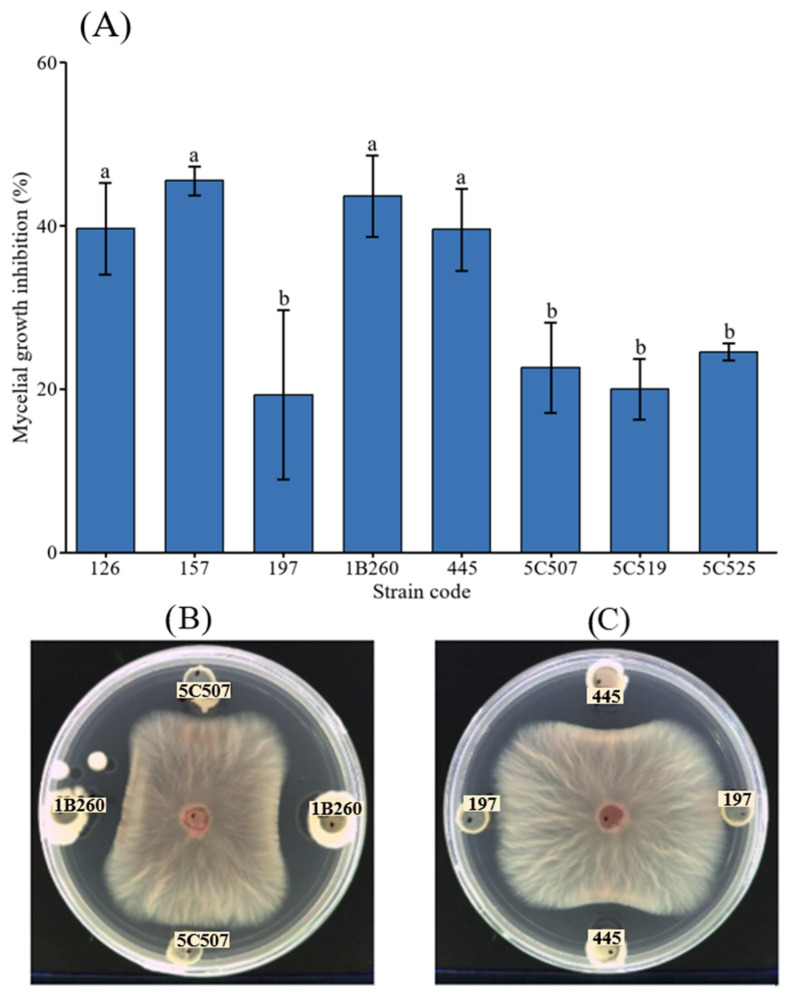
Antifungal activity screening of selected *Streptomyces* strains (**A**) Inhibition percentage of the best eight strains against *Fol*. (**B**) Representative in vitro antagonism assay showing strains 1B260 and 5C507 against *Fol.* (**C**) Representative in vitro antagonism assay showing strains 197 and 445 against *Fol*. Different letters indicate statistically significant differences according to the Tukey test (*p*-value < 0.05); results are presented as mean ± SD (*n* = 3).

The antifungal activity of 70 actinobacterial strains was evaluated through in vitro antagonism assays. Among them, 20 actinobacterial strains exhibited moderate antagonistic activity against *Fusarium oxysporum*, as evidenced by the formation of inhibition zones greater than 20%. For better visualization, Figure 1A shows the eight strains with the highest inhibition percentages among the active isolates. Although several strains showed antifungal activity against *F. oxysporum*, the analysis of mycelial growth inhibition percentages revealed clear differences in their performance as biocontrol agents (Figure 1B,C). Based on these results, strains 1B260, 157, 445, and 126 were selected as the most promising isolates due to their higher and more consistent antifungal activity. No statistically significant differences in *Fol* inhibition were observed among these strains. Among the selected strains, 1B260 showed the highest antifungal activity, achieving 43.5% inhibition of *Fol* mycelial growth (Figure 1A).

#### 2.1.2. Molecular Identification of Strain

All the selected strains mentioned above were isolated and morphologically identified in our previous studies [31]. Based on 16S rRNA sequencing and BLAST+ version 2.17.0 analysis (similarity > 99%), strains 1B260, 157, 126, and 445 were identified as *Streptomyces* sp.

#### 2.1.3. Protease, Pectinase, and Cellulase Activity

The qualitative enzymatic screening revealed that *Streptomyces* strains 126, 157, 445, and 1B260 exhibited protease and cellulase activity, as evidenced by clear hydrolysis zones on skim milk agar and carboxymethylcellulose-amended media, respectively [32].

In contrast, pectinase activity was not detected for either strain under the assay conditions employed, as no visible clearing zones were observed on pectin-containing media. Pectinolytic activity was not detected for strains 126, 157, 445, and 1B260 under the conditions evaluated.

#### 2.1.4. Cytotoxicity Assay of Fermented Supernatants

The cytotoxicity activities of the fermented supernatant produced by the four *Streptomyces* evaluated were determined using 3-(4,5-dimethylthiazol-2-yl)-2,5-diphenyltetrazolium bromide (MTT) assays against HaCaT (Primary Epidermal Keratinocytes) and HDFa (Primary Dermal Fibroblast) cell lines (Table 1).

This assay was conducted as a preliminary screening using cell-free fermentation supernatants. This assay aimed to compare the cytotoxic potential of extracellular metabolites produced under basal fermentation conditions in a more controlled and reproducible approach. The use of viable bacterial cells was avoided, as their continued activity during the assay could complicate interpretation of the results by making it impossible to distinguish whether the observed effect was due to metabolites in the supernatant or to direct interaction of live bacteria with the system under evaluation.

Cultures of strains 1B260 and 445 exhibited minimal cytotoxicity against HaCaT, with half-maximal inhibitory concentrations (IC50) exceeding 1000 ppm. Likewise, strains 1B260 and 445 exhibited low cytotoxicity against the HDFa cell line, with IC50 values greater than 500 ppm. Strain 157 showed the highest cytotoxicity against HaCaT, with an IC_50_ of 98.0 ± 6.2 ppm. On the other hand, strain 126 was the most cytotoxic against HDFa with an IC_50_ of 136.0 ± 2.1 ppm.

#### 2.1.5. Plant Growth Promoter Activities Under In Vitro Conditions

- Nitrogen fixation

Among the four *Streptomyces* strains evaluated, only 1B260 and 445 grew on nitrogen-free Ashby medium, confirming their ability to fix atmospheric nitrogen.

- Phosphate solubilization

**Figure 2:**
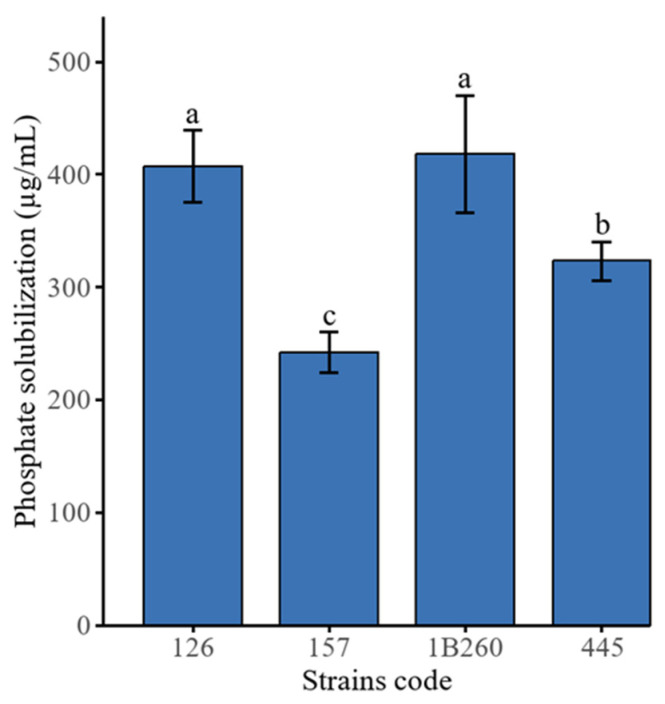
Phosphate solubilization in Pikovskaya liquid broth. Different letters indicate statistically significant differences according to the Tukey–Kramer test (*p*-value < 0.05); results are presented as mean ± SD (*n* = 3).

As shown in Figure 2, the results indicate that phosphate solubilization assays demonstrated strain-dependent differences in the ability to mobilize inorganic phosphorus. Quantitative analysis revealed that strain 1B-260 solubilized 418 µg mL^−1^ of phosphate, whereas strain 445 solubilized 323 µg mL^−1^ indicating a significantly higher phosphorus-mobilizing capacity for 1B-260. (Figure 2).

- Ammonia production

*Streptomyces* strains produced ammonia in peptone water, with concentrations ranging from 1300 to 1400 µg mL^−1^. Statistical analysis indicated no significant differences among strains for this trait (Tukey group [a], *p* > 0.05), suggesting that ammonia production is a shared, conserved plant growth-promoting characteristic among the evaluated isolates.

Based on the preliminary screening of the four candidate strains with greater in vitro inhibition against *Fol*, strains 1B260 and 445 were selected for further evaluation in greenhouse evaluation due to their nitrogen fixation potential, phosphate-solubilizing capacity, and lower cytotoxic activity.

### 2.2. In Vivo Greenhouse Assay

#### 2.2.1. Growth Promotion Assay in Non-Infected Plants

Table 2 summarizes the physicochemical characteristics of the initial soil and the soils after biofertilizer application. The initial soil pH was 5.2 (acidic). The total inorganic nitrogen content was 22.4 mg kg^−1^, primarily in the form of nitrate (20 mg kg^−1^), while the ammonium concentration was 2.4 mg kg^−1^. Available phosphorus was low at 5 mg kg^−1^, and the potassium content was 546 mg kg^−1^.

After treatment application, slight variations were observed in the selected soil parameters reported in Table 2. Soil pH ranged from 5.2 to 5.5. Total inorganic nitrogen was lower in all treatments, particularly in the Streptomyces 445 treatment (3.6 mg kg^−1^). Phosphorus concentrations ranged from 4.0 to 7.0 mg kg^−1^. Table 2 presents selected physicochemical soil properties, whereas the complete characterization, including additional fertility parameters, is provided in the Supplementary Material.

Biofertilizer potential was then assessed by analyzing growth-related variables, including stem length, stem diameter, and number of leaves. Table 3 summarizes the effects of strains 1B260 and 445 on tomato plant growth across the measured variables. Analysis of covariance (ANCOVA) revealed significant differences among the treatments for stem length (*p* < 0.001) and number of leaves (*p* = 0.040). However, no significant effect was detected for stem diameter (*p* = 0.055).

The effects of treatment varied among the evaluated growth variables. Strain 1B260 demonstrated the most favorable response in stem length and was the only treatment to exhibit significant differences. ANCOVA indicated a significant overall effect of treatment on leaf number; however, pairwise comparisons did not identify clear differences among individual treatments. Overall, these results suggest that the effects of the evaluated treatments were strain dependent.

**Figure 3:**
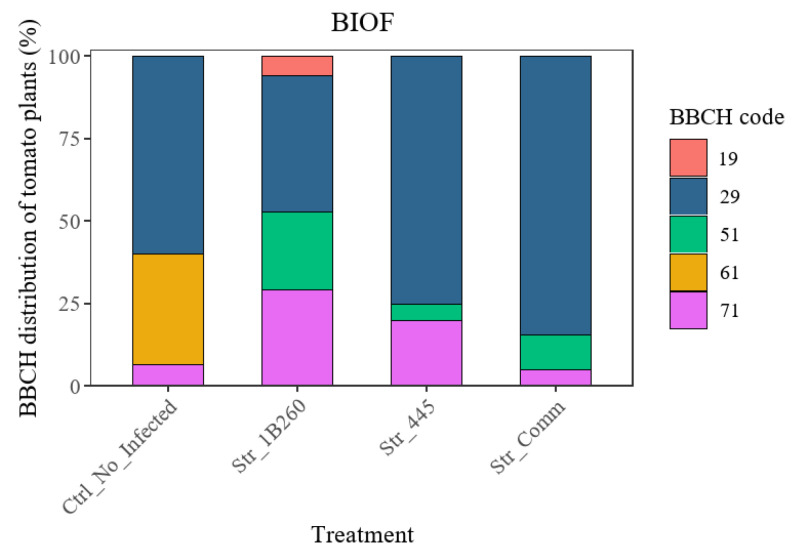
Percentage distribution of tomato plants across BBCH stages: 19 (early vegetative stage), 29 (vegetative stage), 51 (flowering stage), and 71 (early fruit development) under biofertilizer treatments.

As shown in Figure 3, the distribution of tomato plants varied among biofertilizer treatments according to BBCH codes. Codes 19, 29, 51, 61, and 71 are presented as representative categories within the BBCH scale and correspond to the early vegetative stage, vegetative growth, flowering, open flower (anthesis), and early fruit development, respectively. Though individual plants may have reached intermediate BBCH codes, they were grouped by their corresponding developmental phase for clarity.

In BBCH code 29 (vegetative phase), *Streptomyces*-based formulation (Str_Comm) had the highest percentage of plants (84%), followed by *Streptomyces* 445 (75%) and the uninfected control group (60%). *Streptomyces* 1B260 accounted for 47% of the plants at this stage. Additionally, 5.8% of the plants under this treatment were recorded at BBCH code 19 (early vegetative phase). At BBCH code 51 (flowering phase), the non-infected control exhibited the highest percentage (33.3%), followed by *Streptomyces* 1B260 (23%), Str_Comm (10%), and *Streptomyces* 445 (5%). For BBCH code 71 (early fruit development), *Streptomyces* 1B260 exhibited the highest proportion of plants (30%), followed by *Streptomyces* 445 (20%). The non-infected control and Str_Comm presented lower percentages (6.6% and 5.3%, respectively).

**Figure 4:**
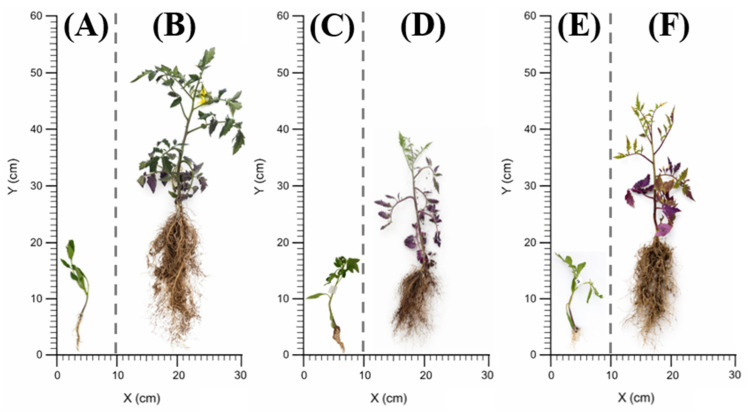
Root system of tomato plants under *Streptomyces* treatments and non-infected control at the beginning and end of the experiment: (**A**) *Streptomyces* 1B260 at the beginning; (**B**) *Streptomyces* 1B260 root after 90 days of the experiment; (**C**) *Streptomyces* 445 at the beginning; (**D**) *Streptomyces* 445 root after 90 days of the experiment; (**E**) non-infected control at the beginning; (**F**) non-infected control root after 90 days of the experiment. Image modified with AI to fix the background and add the scale.

Figure 4 shows representative images of the root system development of tomato plants under *Streptomyces* 1B260, *Streptomyces* 445, and the non-infected control at the beginning and after 90 days of the experiment.

At the beginning of the experiment (Figure 4A), a representative plant treated with *Streptomyces* 1B260 exhibited a total height of approximately 20 cm and a relatively short primary root system measuring about 6 cm. By the end of the experiment, the plants had grown to approximately 55 cm in height. They displayed a markedly expanded root system extending beyond 27 cm in length with greater lateral root density (Figure 4B).

Similarly, a representative plant treated with *Streptomyces* 445 exhibited an initial height of approximately 18 cm (Figure 4C) and a primary root length of about 5 cm. By the end of the experimental period, plant height increased to approximately 39 cm, and the root system extended to roughly 15 cm (Figure 4D). There was a visible increase in branching compared to the initial stage.

In the non-infected control group, the initial plant height was approximately 18 cm, and the root length was approximately 5 cm (Figure 4E). By the end of the experiment, the plants had reached approximately 46 cm in height and had developed a denser root system extending to a depth of approximately 21 cm (Figure 4F).

Overall, the internal comparison among the final-stage plants (B, D, and F) showed that *Streptomyces* 1B260 exhibited the greatest root and shoot development. In contrast, Streptomyces 445 showed a less pronounced growth-promoting response. However, this lower development should not be interpreted as a negative effect, since the statistical analysis showed that strain 445 did not differ significantly from the non-infected control for stem length, stem diameter, or number of leaves. Therefore, the response of strain 445 suggests limited growth promotion compared with strain 1B260, rather than growth inhibition.

#### 2.2.2. Antifungal Assay with *F. oxysporum*—Infected Plants

To assess the antifungal potential of the actinobacterial isolates, their antagonistic activity against *Fusarium oxysporum* was evaluated through in vitro assays. Mycelial growth inhibition was used to compare strain performance and identify the most promising biocontrol candidates.

Table 4 summarizes the effects of various antifungal treatments on the growth of tomato plants, as measured by stem length, stem diameter, and number of leaves. ANCOVA analysis revealed significant differences among the treatments for all three measured parameters (*p* < 0.001 for all).

ANCOVA revealed significant treatment effects on stem length, stem diameter, and number of leaves (Table 4). Overall, pathogen infection was associated with reduced plant growth, particularly regarding stem length and leaf production. The infected control exhibited the least favorable performance across these variables. In contrast, the evaluated treatments partially mitigated these effects; however, their effects varied by strain. Str_Comm had the most favorable effect on stem diameter and, together with strain 445, was also associated with higher leaf production. For stem length, plants treated with *Streptomyces* strains generally performed better than the infected control. However, no significant differences among the treatments with *Streptomyces* strains were observed. Overall, these results suggest that the treatments mitigated the negative effects of infection on tomato growth, with Str_Comm and strain 445 demonstrating the most consistent positive responses across variables.

**Figure 5:**
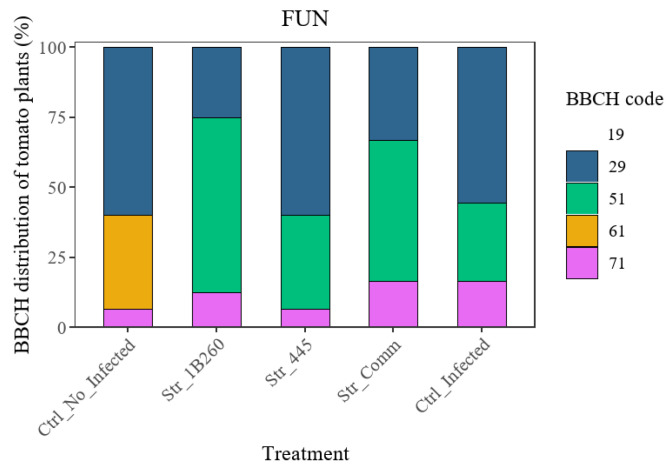
Percentage distribution of tomato plants across BBCH stages 29 (vegetative stage), 51 (flowering stage), 61 (open flower), and 71 (early fruit development) under antifungal treatments.

As shown in Figure 5, the distribution of tomato plants varied by BBCH code among the antifungal treatments. Codes 29, 51, 61, and 71 were presented as representative categories within the BBCH scale and corresponded to vegetative growth, flowering, open flower (anthesis), and early fruit development, respectively.

At BBCH code 29 (the vegetative phase), the highest proportion of plants was observed in the non-infected control and *Streptomyces* 445 (both 60%), followed by the infected control (55.5%). Str_Comm presented at 33.3%, while *Streptomyces* 1B260 exhibited the lowest proportion at this stage (25%). At BBCH code 51 (flowering phase), *Streptomyces* 1B260 exhibited the highest percentage of plants (62.5%), followed by Str_Comm (50%). The non-infected control and *Streptomyces* 445 showed 33.3%, and the infected control showed 27.7%. For BBCH code 71 (early fruit development), both the infected control and Str_Comm exhibited the highest proportion of plants (16.6%), followed by *Streptomyces* 1B260 (12.5%). The non-infected control and *Streptomyces* 445 showed lower percentages (6.6%).

**Figure 6:**
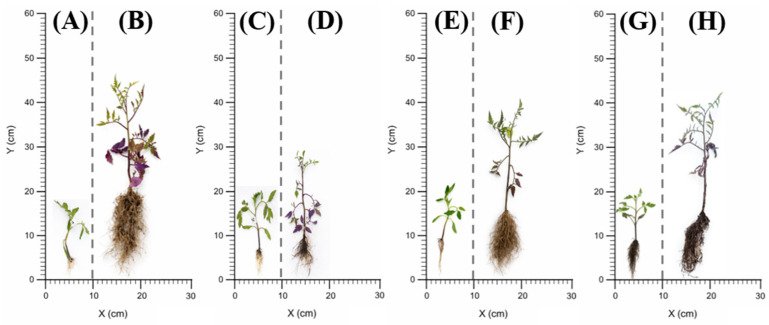
Root system of tomato plants in controls at the beginning and end of the experiment: (**A**) non-infected control at the beginning; (**B**) non-infected control at the end; (**C**) infected control at the beginning; (**D**) infected control after 90 days of the experiment; (**E**) *Streptomyces* 1B260 at the beginning; (**F**) *Streptomyces* 1B260 root after 90 days of the experiment; (**G**) *Streptomyces* 445 at the beginning; (**H**) *Streptomyces* 445 root after 90 days of the experiment. Image modified with AI to fix the background and add the scale.

Figure 6 shows representative images of tomato plant roots at the beginning and end of the experiment (day 90). A calibrated centimeter scale allows for visual estimation of root extensions. The primary root length was approximately 4 cm with sparse lateral branching and low root density. Total root extension from the stem base remained below 5 cm in both treatments. By the end of the experiment, clear differences in root architecture were observed. In the non-infected control (Figure 6B), the root system extended approximately 21 cm from the stem base and developed a dense network of lateral roots, increasing overall root volume.

In contrast, the infected control (Figure 6D) exhibited a comparatively reduced root system with an estimated root extension of 10 cm. Although lateral roots were present, the overall root density and branching were visibly lower than in the non-infected control. Additionally, the total plant height exceeded 40 cm in the non-infected control, whereas the infected control remained below 30 cm.

The *Streptomyces* 1B260 treatment (Figure 6F) showed greater root development than the infected control, with root extension reaching approximately 15 cm and total plant length approximately 41 cm by the end of the experiment. Similarly, the Streptomyces 445 treatment (Figure 6H) showed root extension of approximately 16 cm and total plant length of approximately 42 cm.

## 3. Discussion

This study demonstrated that *Streptomyces* strains exhibiting distinct in vitro potentials can differentially influence tomato plant performance under healthy and pathogen-challenged conditions. The combined evaluation of antifungal activity, plant growth-promoting potential, and cytotoxicity of the supernatants enabled the selection of strains 1B260 and 445 for greenhouse assays, where contrasting functional responses were observed. Strain 1B260 showed a greater capacity to promote plant growth under non-infected conditions, which may be associated with its phosphate-solubilizing ability and nitrogen-related capabilities. In contrast, strain 445 exhibited a greater capacity to maintain plant growth during pathogen infection, suggesting that mechanisms involved in stress mitigation or pathogen suppression may play a more relevant role under biotic stress conditions. These findings highlight the importance of evaluating multifunctional strains under both healthy and infected conditions to better understand their potential agricultural applications.

Of the 70 strains evaluated, only four (1B260, 445, 126, and 157) exhibited significant in vitro antagonistic activity, supporting their selection for further characterization. Molecular identification based on 16S rRNA gene sequencing confirmed their affiliation with the genus *Streptomyces*. The concurrence between antifungal activity and hydrolytic enzyme production suggests a functional relationship in which extracellular proteases and cellulases may contribute to pathogen inhibition by degrading structural components and interfering with fungal development [33]. This is consistent with previous reports indicating that some *Streptomyces* species suppress phytopathogens through the combined action of lytic enzymes, antibiotics, and siderophores [30,34,35]. However, the effectiveness of these mechanisms under in vitro conditions does not necessarily translate into similar outcomes in planta.

In addition to its well-documented biocontrol potential, the genus *Streptomyces* has been widely recognized for its role as a biofertilizer through multiple growth-promoting mechanisms [36,37,38]. In this context, the present study extends previous findings by simultaneously evaluating both the antifungal activity and growth-promoting effects of *Streptomyces* strains 1B260 and 445 in tomato plants under greenhouse conditions, providing a more integrated assessment of their agronomic potential. Within this context, the in vitro inhibition levels observed in this study are consistent with previous reports, supporting the biocontrol potential of the selected strains [35,39,40].

Previous studies have reported in vitro inhibition levels of up to 55.56% against *F. oxysporum* [35]. The inhibition levels observed in this study are consistent with those reported in this study, supporting the classification of the selected strains as potential biocontrol agents. However, integrating antifungal activity, enzyme production, growth-promoting potential, and cytotoxicity profiles provided a more robust basis for strain selection. The enzymatic activity (proteases, cellulases, and pectinases) contributes to pathogen inhibition through the degradation of structural components [41]. The superior phosphate-solubilizing capacity of strain 1B260, the highest among the evaluated strains, suggests that phosphorus mobilization may have played an important role in its plant growth-promoting effect and supported its selection for subsequent in vivo assays [38].

Cytotoxicity was assessed using cell-free fermentation supernatants solely as a preliminary evaluation of extracellular metabolites. This assay does not reflect the behavior or safety of live *Streptomyces* cells after soil inoculation, in which biocontrol activity is influenced by interactions among the bacterium, the pathogen, the plant, and the native microbiota [42]. The lower cytotoxicity observed for strains 445 and 1B260 supported their selection for in vivo evaluation. Nevertheless, further toxicological, genomic, and metabolic analyses would be required to validate their safe use as agricultural bio-inputs (Resolución No. 068370, 2020) [43].

Accordingly, *Streptomyces* strains 445 and 1B-260 were identified as the most promising candidates and were selected for in vivo evaluation. Under greenhouse conditions, the effects of these strains were strongly dependent on their functional attributes, revealing distinct patterns in plant growth responses. The treatments significantly influenced both vegetative and reproductive development in tomato plants, although the magnitude and direction of the response were strain dependent. Strain 1B260 showed a pronounced effect on stem elongation. In contrast, strain 445 exhibited a comparatively lower effect on plant height but was associated with a more stable response in leaf production and stem diameter under infected conditions. In contrast, the commercial formulation (Str_Comm) showed a more uniform but less pronounced effect across the evaluated variables.

Despite these positive effects on plant growth, biofungicidal activity was more evident under in vitro assays than under greenhouse conditions. This outcome is consistent with the observations of Bonaterra [22], who reported that biocontrol efficacy often decreases in complex pathosystems due to simultaneous interactions among the plant, soil, microorganisms, and pathogen. Under in vivo conditions, Str_Comm and *Streptomyces* 445 showed greater agronomic improvements than the infected control, particularly in leaf number. This behavior is consistent with previous studies reporting that some *Streptomyces* can mitigate the impact of pathogens during plant growth. For instance, Zeyad [36] evaluated *Streptomyces araujoniae* strains (TN11 and TN19) in chickpea under greenhouse conditions, showing a 50–58% reduction in *Fusarium oxysporum* f. sp. ciceris severity and significant improvements in growth parameters. Similarly, Khan [30] reported the effectiveness of several *Streptomyces* strains (*Streptomyces* sp. JCK-6131, *Streptomyces* strain CACIS-1.5CA, *Streptomyces griseus* H7602, *Streptomyces griseorubens* E44G, *Streptomyces* strain 22-4) against *Fol* and other pathogens in tomato (*Solanum lycopersicum*), highlighting their roles in both disease suppression and plant growth promotion.

Under *F. oxysporum* infection, the differences between treatments were comparable to those reported in the literature. The reduction in growth in the infected control agrees with the classic symptoms of vascular wilt, characterized by decreased vigor, foliar reduction, and impaired vegetative development [44,45,46,47]. In this context, strain 1B260 showed the most favorable response in stem length, while 445 and the commercial Str_Comm exhibited intermediate effects in the stem length variable. This pattern is consistent with studies by Abbasi [46] and Kanini [48], which reported increased tomato plant height following the application of specific *Streptomyces* strains under soil-borne pathogen stress. Notably, the concentration used by Abbasi [46] (10^6^ CFU g^−1^) is comparable to that applied in this study, whereas Kanini [49] employed a higher concentration (10^9^ spores mL^−1^) via seed coating, suggesting that growth-promoting effects can be achieved across different application strategies [49].

Likewise, the higher total leaf count observed in treatments such as Str_Comm and *Streptomyces* 445, compared to the infected control, agrees with previous studies reporting that beneficial microorganisms can reduce the severity of foliar symptoms caused by *Fusarium* infection. Proposed mechanisms include antibiosis, induced systemic resistance, or the production of lytic enzymes [30,50,51]. Nevertheless, further studies are needed to elucidate the mechanism underlying the biocontrol activity of *Streptomyces* 445.

Concurrently, the results evidenced clear biofertilizer activity, particularly in strain 1B260, which promoted stem elongation and root extension (≈24 cm), outperforming the non-infected control. Prior studies indicate that different isolates from *Streptomyces* spp. (*Streptomyces* sp. strain HM2, *Streptomyces thinghirensis* strain HM3, *Streptomyces* sp. strain HM8, and *Streptomyces tricolor* strain HM10) function as plant growth promoters, exerting more pronounced effects on stem elongation and various vegetative characteristics [38,52].

However, the effects were not uniform across variables or strains. While 1B260 showed a clear positive effect on stem elongation, strain 445 exhibited a stronger effect on leaf number. This agrees with previous evidence for microbial biofertilizers, where the response depends on the strain and its specific interaction with the plant and environment, and cannot be directly extrapolated [22,53,54]. The greater elongation observed in plants treated with strain 1B260 could be partially associated with its superior phosphate-solubilizing capacity, which was the highest among the evaluated strains. Increased phosphorus availability in the rhizosphere has been reported to contribute to energy transfer, root development, and nutrient acquisition, thereby potentially supporting vegetative growth and plant elongation [55,56,57].

Regarding soil physicochemical conditions, the primary variations associated with *Streptomyces* inoculation were observed in nitrogen content, particularly in the ammonium (NH_4_^+^) and nitrate (NO_3_^−^) fractions, as well as total nitrogen. Strain 1B260 presented the highest values for NH_4_^+^ (5.5 mg kg^−1^), NO_3_^−^ (6.2 mg kg^−1^), and total nitrogen (11.7 mg kg^−1^) compared to the control and strain 445, suggesting a greater influence on soil nitrogen dynamics. These results are consistent with those reported by Peng [58], who demonstrated that *Streptomyces* JD211 strain can mediate the transformation of nitrogen into more bioavailable forms for plants [58]. These differences in nitrogen availability may be related to the biofertilizer effects observed in vegetative growth, particularly the stem elongation promoted by strain 1B260. The increased availability of mineral nitrogen is associated with enhanced agronomic performance, consistent with studies linking higher nitrogen availability to improved vegetative growth [59].

The phenological distribution (BBCH codes) suggests that plant performance is determined by developmental regulation rather than by biomass accumulation [60]. The infected control showed a higher proportion of plants at BBCH 71, whereas the non-infected control had a greater representation at BBCH 61, indicating more sustained flowering and greater potential reproductive capacity [60].

Under non-stress conditions, strain 1B260 promoted a more advanced progression toward reproductive stages, which may be partially associated with its phosphate-solubilizing capacity and nitrogen-related plant growth-promoting activities. Enhanced phosphorus availability has been reported to contribute to reproductive development and fruit formation in tomato, potentially favoring an earlier transition from vegetative to reproductive stages [61,62]. However, because phenological development is influenced by multiple interacting factors that were not specifically controlled for or quantified in this study, these factors may have contributed to the observed phenological response rather than representing a direct causal relationship. In contrast, plants treated with strain 445 remained predominantly in vegetative stages and showed slower progression toward reproductive development, which may reflect a distinct growth-promoting pattern focused on vegetative development rather than accelerated reproductive progression.

Under *Fol* infection, the infected control exhibited a disruption of vegetative development, consistent with xylem colonization and altered assimilate transport [63]. In this context, strain 1B260 showed the greatest capacity to maintain phenological progression toward flowering, suggesting a partial mitigation of the pathogen’s effect. Meanwhile, strain 445 and *Streptomyces*-based commercial formulation exhibited intermediate responses, indicating a limited capacity to sustain development under stress conditions. This is consistent with previous reports indicating that some *Streptomyces* strains, such as *Streptomyces malaysiensis* MJM1968 and *Streptomyces tendae* F4, can mitigate the impact of biotic stress at different stages, including the vegetative and reproductive stages [30,64].

Finally, the morphological observations are consistent with the phenological patterns described above. Plants treated with strain *Streptomyces* 1B260 showed a more developed and spread-out root system than the controls. This indicates that the treatment influenced root development, which likely contributed to the observed effects on aerial growth. This response is particularly relevant, as *Fusarium* infection is known to reduce root elongation and the capacity for soil exploration [65,66]. Collectively, the consistency between root structure and phenological progression supports the hypothesis that the observed changes reflect a coordinated regulation of plant development rather than a random increase in growth.

## 4. Materials and Methods

### 4.1. In Vitro Assay

#### 4.1.1. Strains and Culture Conditions

Previous studies have identified various Streptomyces strains isolated from the Guaviare and Arauca rivers in Colombia and reported their potential biocontrol activity against various pathogenic bacteria and fungi [32,67]. Based on these findings, 70 strains were selected for further investigation based on their reported major biological activity. The strains were reactivated on ISP2 (International *Streptomyces* Project Medium 2) agar plates using the spread plate method and incubated for 7 days at 30 °C to ensure optimal sporulation [32]. Morphological characterization was conducted both macroscopically and microscopically in accordance with the guidelines of the International *Streptomyces* Project (ISP). Molecular identification was conducted by PCR amplification and sequencing of the nearly complete 16S rRNA gene. The locus was amplified using primers 27F (5′-AGAGTTTGATCMTGGCTCAG-3′) and 1492R (5′-TACGGYTACCTTGTTACGACTT-3′), following previously described thermal cycling conditions. PCR products were purified and sequenced by Macrogen (Seoul, Republic of Korea), and the resulting sequences were compared against reference strains available in the NCBI GenBank database to confirm their taxonomic identity [31].

In parallel, *Fol* was kindly provided by the Biotechnology and Genetics Research Group of Universidad Colegio Mayor de Cundinamarca (Bogotá, Colombia). The fungus was cultured on potato dextrose agar (PDA) and Sabouraud agar supplemented with chloramphenicol to prevent bacterial contamination [68]. Cultures were incubated at 30 °C and periodically subcultured to ensure viability and purity for subsequent use in both in vitro and in vivo assays.

#### 4.1.2. Antagonistic Assay Against the Pathogen

*Streptomyces* strains were evaluated for their antagonistic biological activity using a dual-culture assay. A 9 mm diameter plug was taken from a 7-day-old culture of *Streptomyces* and phytopathogens; the phytopathogen plug was extracted from the central region of a predominantly sporulated colony. The Streptomyces plug was then placed at an equal distance from the center towards the periphery of the plate, 2.5 cm from the phytopathogen plug placed in the center of the PDA plates, using 90 × 15 mm Petri dishes. After this, all plates were incubated for 7 days at 25 °C. *Fol* cultures without *Streptomyces* were used as a negative control. The percentage of inhibition (*PI*) of the fungus was calculated according to Equation (1) established by Shahid [69]:

(1) $$PI=\frac{R-R_{i}}{R}\times 100$$

where *R* corresponds to the radial growth of the phytopathogen in the control treatment, and *R*_i_ represents the radial growth of the phytopathogen in the presence of *Streptomyces*.

#### 4.1.3. Molecular Identification of Strains

The four *Streptomyces* strains with the highest antifungal activity and compatibility were selected and identified by 16S rRNA gene sequencing. The universal primers 27F and 1492R were used to amplify 16S rRNA from genomic DNA. Amplification was performed in a total volume of 50 μL using 125 ng of genomic DNA as a template, 2X PCR Master Mix containing Taq DNA polymerase, (Thermo Fisher Scientific, Waltham, MA, USA), and 10 μM of each primer. PCR conditions were maintained as described by Khadayat [70].

#### 4.1.4. Protease, Pectinase, and Cellulase Activity

Protease, pectinase, and chitinase production were evaluated using skim milk agar, M9 minimal salt amended with pectin (1% *w*/*w*), and M9 minimal salt Carboxymethylcellulose (CMC) agar, respectively [70,71,72]. Active colonies of the fourth active *Streptomyces* strain were inoculated onto each agar plate and incubated for 7 days at 30 °C. Colonies with a clear halo zone in each agar were identified as enzyme producers.

#### 4.1.5. Cytotoxicity Assay of Supernatants

The cytotoxic effect of a 7-day fermented supernatant was evaluated in human primary dermal fibroblasts (HDFa, ATCC^®^ PCS-201-012™, Primary Dermal Fibroblasts; Normal, Human, Adult) and Adult Human Keratinocytes with Low Calcium and High Temperature (HaCaT, A11.251.210.353, DeCS/MeSH). Both cell lines were cultured in Dulbecco’s Modified Eagle Medium (DMEM) supplemented with 10% fetal bovine serum at 37 °C and 5% CO_2_. Cells were seeded into a 96-well plate at a density of 2 × 10^4^ cells/well and incubated overnight before treatment exposure. The cells were treated for 24 h with microbial fermented supernatant at 1000, 500, 100, and 10 μg mL^−1^. Dimethyl sulfoxide (DMSO, 2.5–10% *v*/*v*) was used as the positive control. After treatments, supernatants were replaced with fresh medium containing MTT at 0.5 mg mL^−1^, and the cells were incubated for 4 h [73]. Then, the MTT was removed, and DMSO was added to each well to dissolve formazan crystals. The amount of formazan was measured by its absorbance at 570 nm. Cell viability is calculated using Equation (2):

(2) $$Cell\;viability\;\left(\%\right)\;=\;1-\;\frac{A_{T}}{A_{U}}\times 100$$

where A_T_ corresponds to the absorbance of treated cells, and A_U_ is the absorbance of untreated cells.

#### 4.1.6. Plant Growth Promoter Activities

- Nitrogen fixation

To confirm nitrogen fixation, each *Streptomyces* strain was inoculated into Ashby medium (nitrogen-free culture medium) and incubated for 7 days at 30 °C, as described by Li [74], with some modifications. Colony formation was taken as nitrogen-fixing *Streptomyces*. Ashby medium composition per liter was glucose (10 g), dipotassium phosphate (0.2 g), magnesium sulfate (0.2 g), sodium chloride (0.2 g), potassium sulfate (0.1 g), calcium carbonate (5.00 g), and bacteriological agar (15.00 g).

- Phosphate solubilization

First, a qualitative method was used to test phosphate solubilization activities. Each strain was inoculated onto Pikovskaya’s agar medium as described by Balakrishnan [75]. Positive strains were identified by the presence of a clean halo zone around the colonies and were further used to quantify phosphate solubilization. A quantitative method was used to estimate phosphate solubilization. Initially, 25 mL of Pikovskaya liquid broth was inoculated with each *Streptomyces* strain and incubated for 7 days at 30 °C in a shaking incubator at 150 rpm (TECNAL, Piracicaba, Brazil). Then, the supernatant was collected by centrifugation for 10 min at 8000 rpm (Fisher Scientific, Waltham, MA, USA, EE. UU). Finally, phosphate was measured using microtiter plates with 100 µL of supernatant mixed with 12.5 µL of ammonium molybdate in 1N sulfuric acid and 12.5 µL of 10% of ascorbic acid. The absorbance was measured at 650 nm in a multimode reader (Varioskan, Thermo Scientific TM, Waltham, MA, USA, EE. UU) [76,77]. The quantitative phosphate solubilization value was calculated using a standard curve for monopotassium phosphate and expressed in µg/mL.

- Ammonia production

*Streptomyces* strains were inoculated into 3 mL of peptone water (1% *w*/*v*) and incubated at 28 °C at 150 rpm for 7 days (TECNAL, São Paulo, Brazil). After incubation, the supernatant was collected by centrifugation for 10 min at 8.000 rpm (Fisher Scientific TM, EE. UU). Subsequently, 190 µL of cell-free culture was mixed with 9.5 µL of Nessler’s reagent using microtiter plates. Ammonia production was validated by the formation of a dark yellow to brown color. Then, absorbance was measured at 530 nm in a multimode reader (Varioscan, Thermo Scientific TM, EE. UU.) and interpolated from a standard curve of ammonium sulfate and expressed in mg/mL [78,79].

Based on the in vitro screening, strains 445 and 1B260 were selected for the greenhouse assays. This selection was primarily based on their lower cytotoxicity, with IC_50_ values > 500 µg/mL for strain 445 and >1000 µg/mL for strain 1B260 across the evaluated cell lines. Additionally, both strains showed plant growth–promoting activities under in vitro conditions, supporting their selection for subsequent in vivo evaluation.

### 4.2. In Vivo Greenhouse Assay

#### 4.2.1. Tomato Seedlings

A total of 200 one-month-old seedlings of *Solanum lycopersicum* Mill. cv. Chonto (Chonto tomato) were obtained from the Fundación par a el Desarrollo Universitario (Carretera Central del Norte km 3, via La Caro, Chía, Cundinamarca, Colombia). At the time of purchase, seedlings of uniform size and without visible phytosanitary symptoms were used in the in vivo assays conducted in a greenhouse. Before the application of the experimental treatments, all seedling roots were separated from the original nursery substrate, washed with tap water, immersed in 70% ethanol for 1 min, treated with sodium hypochlorite at 200 ppm for 1 min, and subsequently rinsed three times with sterile tap water to reduce potential biases associated with the native nursery microbiota [80]. Transplanting was carried out after soil pretreatment, as described below, and the seedlings were immediately subjected to the corresponding experimental treatments to evaluate the plant’s growth-promoting and antifungal effects.

#### 4.2.2. Greenhouse Conditions

All plants were maintained under greenhouse conditions throughout the experiment. The average morning and afternoon temperatures were 21.7 ± 2.5 °C and 29.9 ± 5.3 °C, respectively, with relative humidity ranging from 31% to 59%. Plants were exposed to natural daylight conditions from June to September 2025. Irrigation was performed using tap water as required to maintain constant soil moisture.

#### 4.2.3. Growth Promotion Assay (Non-Infected Plants)

To evaluate plant growth–promoting effects, a total of 80 seedlings grown in 750 g of soil were divided into four treatment groups, each consisting of 20 replicates: (i) *Streptomyces* strain 445, (ii) *Streptomyces* strain 1B260, (iii) a commercial *Streptomyces*-based formulation (Str_Comm), and (iv) a water-treated control. Therefore, the number of replicates included in the statistical analysis is reported as *n* in each corresponding results table, excluding plants that did not survive the first week after transplanting. Treatments were applied via direct soil inoculation in the rhizosphere, using a pipette to dispense 1 mL per plant of a suspension containing 1.0 × 10^6^ spores mL^−1^ of the corresponding *Streptomyces* strain. Spore suspensions were prepared in sterile 0.85% saline solution by scraping the sporulated mycelium from 7-day-old cultures grown on PDA plates. The spore concentration was quantified using a Neubauer chamber and adjusted to the required concentration of 1.0 × 10^6^ spores mL^−1^ by dilution when necessary [81]. Applications were performed on days 1, 7, 14, 21, and 28 after transplanting, followed by applications every 15 days on days 42, 56, 70, 84, and 98. Str_Comm was applied according to the manufacturer’s instructions (5 mL of the commercial formulation per liter of water), delivering 50 mL of the prepared solution per plant on the same days already described above.

For seedlings subjected to treatments with *Streptomyces* strains 445 and 1B260, the soil was pretreated one week before transplanting by inoculation with the selected strains at a concentration of 1.0 × 10^6^ spores mL^−1^. After inoculation, the soil was maintained without irrigation or additional inputs until transplanting seven days after the pre-treatment.

##### Physicochemical Characterization

Soil characterization was performed using an initial composite soil sample and final composite samples for each treatment. For each treatment, soil subsamples were randomly collected from the rhizosphere zone of multiple plants by taking soil portions from different locations within each experimental unit. Subsequently, the subsamples were thoroughly homogenized to obtain one composite sample per treatment. This approach was used to evaluate potential changes in soil physicochemical properties associated with applied treatments.

Therefore, the physicochemical characterization of these composite samples was carried out at the Fundación para el Desarrollo Universitario (Chía, Cundinamarca, Colombia). A partial chemical fertility analysis, including organic matter determination, was conducted. The analyzed parameters included soil reaction (pH), electrical conductivity (EC), cation exchange capacity (CEC), effective cation exchange capacity (ECEC), permanent wilting point (PWP), soil saturation percentage (SP), organic carbon, organic matter (OM), mineral nitrogen (N-mineral: NH_4_^+^ + NO_3_^−^), available phosphorus (P), exchangeable potassium (K^+^), calcium (Ca^2+^), magnesium (Mg^2+^), available sulfur (S), exchangeable sodium (Na^+^), chloride (Cl^−^), exchangeable aluminum (Al^3+^), and cationic ratios (Ca/Mg, Ca/K, Mg/K), which are commonly used to define soil quality [82].

#### 4.2.4. Antifungal (Biocontrol) Assay (*Fol*—Infected Plants)

To evaluate antifungal activity, a total of 100 seedlings grown in 750 g of soil were divided into six treatment groups, each consisting of 20 replicates: (i) *Streptomyces* strain 445, (ii) *Streptomyces* strain 1B-260, (iii) Str_Comm, (iv) a water-treated control, and (v) a pathogen-infected control.

Treatments containing *Streptomyces* strains (445 and 1B260) were applied via direct soil inoculation in the rhizosphere, using a pipette to dispense 1 mL per plant of a suspension containing 1.0 × 10^6^ spores mL^−1^ of the corresponding strain. Applications were performed on days 1, 7, 14, 21, and 28 after transplanting. Finally, Str_Comm was applied according to the manufacturer’s recommendations, and the seedlings were inoculated with *Fol* as described in Section Inoculation.

##### Inoculation

For the antifungal assays, seedling roots underwent a surface disinfection protocol prior to pathogen inoculation. Roots were washed with tap water, immersed in 70% ethanol for 1 min, treated with sodium hypochlorite at 200 ppm for 1 min, and subsequently rinsed three times with sterile tap water. Pathogen inoculation was carried out by immersing the roots in a *Fol* spore suspension at a concentration of 1.0 × 10^6^ spores mL^−1^, following reported methodologies [83,84], and then they were planted in the soil according to the treatment.

#### 4.2.5. Measurements of Agronomic and Growth Parameters

For the two in vivo trials, agronomic and growth parameters were measured over a 98-day experimental period. The variables evaluated included the number of leaves, height, and stem diameter, the latter measured with a digital caliper. Measurements were recorded on days 7, 14, 21, and 28 after transplanting, and subsequently every 15 days on days 42, 56, 70, 84, and 98.

### 4.3. Statistical Analysis

All statistical analyses were performed using R software (v4.5.1). Treatment effects on plant growth variables (number of leaves, stem length, and stem diameter) were evaluated using analysis of covariance (ANCOVA), including the initial measurement as a covariate. Significant differences (*p* < 0.05) were assessed using Tukey’s HSD test. Results are expressed as adjusted means ± standard deviation (SD).

## 5. Conclusions

This study demonstrated that *Streptomyces* strains (1B260 and 445) can function as multifunctional agents in tomato production by combining antifungal activity against *Fusarium oxysporum* f. sp. *lycopersici* (*Fol*) with plant growth-promoting properties. However, the beneficial effects varied with strain identity and plant health status. Under non-stress conditions, strain 1B260 significantly promoted plant growth, particularly stem elongation, and plants treated with this strain also showed a more advanced progression toward reproductive stages relative to other treatments. These effects may be associated with its superior in vitro phosphate-solubilizing capacity and nitrogen-related activities, potentially improving nutrient availability in the rhizosphere. In contrast, strain 445 showed a greater capacity to preserve plant growth under pathogen pressure, suggesting a partial contribution to stress mitigation than to direct growth stimulation.

Overall, under the greenhouse conditions evaluated, growth promotion was more consistently observed than direct pathogen suppression, highlighting the potential relevance of plant-mediated and nutritional contributions to the performance of microbial inoculants. These results support the strategic selection of *Streptomyces* 1B260 and 445 strains as promising inoculants with complementary functions for integrated biofertilization and biocontrol in tomato production. However, further studies are needed to validate strain performance under field conditions and optimize inoculation strategies, including application frequency and concentration. Additional research should also address rhizosphere microbiota interactions, root colonization dynamics, and mechanisms underlying the observed plant responses.

## Figures and Tables

**Table 1:** IC_50_ values (ppm) of fermented supernatants from *Streptomyces* strains against HaCaT (human keratinocyte) and HDFa (human dermal fibroblast adult) cell lines using MTT assays. Values are presented as mean ± SD (*n* = 3).

*Streptomyces* Supernatant	IC_50_ Against HaCaT Cell Line (ppm)	IC_50_ Against HDFa Cell Line (ppm)
**126**	210 ± 6.5	136 ± 2.1
**157**	98 ± 6.2	248 ± 7.9
**445**	>500	>1000
**1B260**	888 ± 3.5	>1000

**Table 2:** Physicochemical characterization of the initial soil and final composite soil samples after biofertilizer treatment application. Soil macronutrients are expressed as mg kg^−1^.

		Soil Macronutrients (mg/kg^−1^)				
Sample	pH	Ammonium (NH_4_^+^)	Nitrate (NO_3_^−^)	Total Inorganic Nitrogen	Phosphorus	Potassium
**Initial_soil**	5.2	2.4	20	22.4	5.0	546
**Ctrl_No_Infected**	5.3	4.9	2.1	7.0	7.0	492
**Str_445**	5.5	2.9	0.7	3.6	5.0	452
**Str_1B260**	5.2	5.5	6.2	11.7	4.0	463

Note: Values correspond to one composite sample per treatment analyzed by an external laboratory and are reported according to the decimal precision provided in the analytical report.

**Table 3:** Effects of biofertilizer treatments on stem length (cm), stem diameter (mm), and number of leaves in tomato plants. Values are presented as final means ± SD. Different letters indicate significant differences according to Tukey’s test (*p* < 0.05). ANCOVA *p*-values were < 0.001 (stem length), 0.055 (stem diameter), and 0.0406 (number of leaves). Ctrl_No_Infected: non-infected control; Str_Comm: commercial *Streptomyces*-based formulation. The final number of plants per treatment (n) varied because plants that did not survive the first week after transplanting were excluded from the final analysis.

Treatment	n	Stem Length		Stem Diameter		Number of Leaves	
		Final Mean ± SD (cm)	Tukey	Final Mean ± SD (mm)	Tukey	Final Mean ± SD	Tukey
**Ctrl_No_Infected**	15	25.1 ± 2.0	a	4.46 ± 0.31	a	40.0 ± 7.4	a
**Str_1B260**	17	27.8 ± 4.1	b	4.43 ± 0.54	a	38.8 ± 6.2	a
**Str_445**	20	26.2 ± 2.5	a	4.47 ± 0.56	a	34.7 ± 7.1	a
**Str_Comm**	18	24.4 ± 4.2	a	4.42 ± 0.38	a	38.7 ± 7.8	a

**Table 4:** Effects of antifungal treatments on stem length (cm), stem diameter (mm), and number of leaves in tomato plants. Values are presented as final means ± SD. Different letters indicate significant differences according to Tukey’s test (*p* < 0.05). ANCOVA *p*-values were <0.001 (stem length), <0.001 (stem diameter), and <0.001 (number of leaves). Ctrl_Infected: infected control; Ctrl_No_Infected: non-infected control; Str_Comm: commercial *Streptomyces*-based formulation. The final number of plants per treatment (n) varied because plants that did not survive the first week after transplanting were excluded from the final analysis.

Treatment	n	Stem Length		Stem Diameter		Number of Leaves	
		Final Mean ± SD (cm)	Tukey	Final Mean ± SD (mm)	Tukey	Final Mean ± SD	Tukey
**Ctrl_No_Infected**	15	24.2 ± 1.8	bc	4.24 ± 0.28	d	40.0 ± 7.4	ab
**Ctrl_Infected**	18	23.6 ± 2.1	c	4.41 ± 0.36	b	28.5 ± 4.7	c
**Str_1B260**	16	24.6 ± 2.5	ab	4.29 ± 0.44	cd	30.7 ± 8.0	bc
**Str_445**	15	24.4 ± 4.1	ab	4.31 ± 0.39	bc	33.6 ± 6.1	a
**Str_Comm**	18	24.6 ± 2.2	a	4.49 ± 0.46	a	40.0 ± 5.5	a

## Data Availability

The data presented in this study is available on request from the corresponding author.

## Supplementary Materials

The following supporting information can be downloaded at https://www.mdpi.com/article/10.3390/plants15121766/s1. Table S1 Physicochemical characterization of the initial soil and the final soils after the application of biofertilizer treatments.
