# Supplementary material for: Biofertilizer and Antifungal Potential of Streptomyces spp. in Greenhouse-Grown Tomato Plants (Solanum lycopersicum Mill.)
Source: Plants (Basel). 2026 Jun 8;15(12):1766. doi: 10.3390/plants15121766 (PMC13306408; doi:10.3390/plants15121766)
Supplement: Supplementary file 1 [file plants-15-01766-s001.zip › plants-4320757-supplementary.pdf]

Table S1. Physicochemical characterization of the initial soil and the final soils after the application of biofertilizer treatments

			Soil macronutrients (mg kg^{-1})										cmol (+)* Kg	Cationic relations				Other analyses and sample parameters					
Sample	pH	E.C. dS/m	Ammonium (NH_4^+)	Nitrate (NO_3^-)	Total Inorganic Nitrogen	Phosphorus	Potassium	Calcium	Magnesium	Sodium	Sulfur	Chloride	Acidity	Ca/Mg	Ca/K	Mg/K	(Ca+Mg)/K	% Water	% Saturation	% Organic Carbon	% Materia Orgánica	CEC (cmol $^+$ *Kg $^{-1}$)	ECEC (cmol $^+$ *Kg $^{-1}$)
Initial soil	5,2	0,71	2,4	20	22,4	5	546	1051	171	34	29,6	97	0,18	3,73	3,76	1,01	4,76	6,2	95,6	5,84	10,04	45,9	8,4
Ctrl_No_Infected	5,3	0,64	4,9	2,1	7	7	492	1057	166	53	31,1	118	0,15	3,86	4,19	1,09	5,27	5	89,3	5,65	9,72	43,9	8,3
Str_445	5,5	0,51	2,9	0,7	3,6	5	452	1049	151	70	35,4	86	0,15	4,22	4,52	1,07	5,59	7	96,3	5,63	9,68	44,3	8,1
Str_1B260	5,2	0,69	5,5	6,2	11,7	4	463	1232	162	76	21,8	118	0,14	4,61	5,19	1,13	6,32	5,2	84,1	5,79	9,96	43,8	9,1
